# Recurrent Pneumothorax in a Critically Ill Ventilated COVID-19 Patient

**DOI:** 10.1155/2020/8896923

**Published:** 2020-09-18

**Authors:** Lucas Rehnberg, Robert Chambers, Selina Lam, Martin Chamberlain, Ahilanandan Dushianthan

**Affiliations:** ^1^General Intensive Care Unit, University Hospital Southampton NHS Foundation Trust, Southampton, Tremona Road, Southampton SO16 6YD, UK; ^2^Cardiothoracic Radiology, University Hospital Southampton NHS Foundation Trust, Southampton, Tremona Road, Southampton SO16 6YD, UK; ^3^Department of Thoracic Surgery, University Hospital Southampton NHS Foundation Trust, Southampton, Tremona Road, Southampton SO16 6YD, UK

## Abstract

We present this case of a young woman with SARS-CoV-2 viral infection resulting in coronavirus 2019 (COVID-19) lung disease complicated by a complex hydropneumothorax, recurrent pneumothorax, and pneumatoceles. A 33-year-old woman presented to the hospital with a one-week history of cough, shortness of breath, and myalgia, with no other significant past medical history. She tested positive for COVID-19 and subsequently, her respiratory function rapidly deteriorated, necessitating endotracheal intubation and mechanical ventilation. She had severe hypoxic respiratory failure requiring a protracted period on the mechanical ventilator with different ventilation strategies and multiple cycles of prone positioning. During her proning, after two weeks on the intensive care unit, she developed tension pneumothorax that required bilateral intercostal chest drains (ICD) to stabilise her. After 24 days, she had a percutaneous tracheostomy and began her respiratory wean; however, this was limited due to the ongoing infection. Thorax CT demonstrated a left-sided pneumothorax, with bilateral pneumatoceles and a sizeable, complex hydropneumothorax. Despite the insertion of ICDs, the hydropneumothorax persisted over months and initially progressed in size on serial scans needing multiple ICDs. She was too ill for surgical interventions initially, opting for conservative management. After 60 days, she successfully underwent a video-assisted thoracoscopic surgery (VATS) for a washout and placement of further ICDs. She was successfully decannulated after 109 days on the intensive care unit and was discharged to a rehabilitation unit after 116 days of being an inpatient, with her last thorax CT showing some residual pneumatoceles but significant improvement. Late changes may mean patients recovering from the COVID-19 infection are at increased risk of pneumothoracies. Clinicians need to be alert to this, especially as bullous rupture may not present as a classical pneumothorax.

## 1. Introduction

Clinical investigation with computed tomography (CT) scanning is ubiquitous in modern medicine, and COVID-19 is not an exception. There have been several cohort studies investigating the radiological changes in patients with COVID-19 lung disease [1, 2]. CT is recommended in suspected COVID-19 cases to help guide clinical management in those patients with severe respiratory distress, those who deteriorate clinically, and to help deal with possible complications.

The most common CT findings are bilateral ground-glass opacification (GGO) (87.5-88%) and consolidation (31.8%) with peripheral (76%) or multilobar involvement (78.8%), most commonly seen within the first two weeks of the illness. There are now a handful of case reports of spontaneous pneumothorax and pneumomediastinum in the COVID-19 population [3–9]. However, these case reports are found in patients who only require supplementary oxygen on the wards or noninvasively ventilated (NIV) patients. We present a rare case of ventilated patient suffering with recurrent tension pneumothoracies and complex infected hydropneumothorax with pneumatoceles, in addition to the classical COVID-19 changes, as a complication of the COVID-19 infection.

## 2. Case Presentation

A 33-year-old woman presented with a one-week history of cough, shortness of breath, and myalgia, with no other significant past medical history except depression and an ectopic pregnancy in 2019 requiring salpingectomy. Otherwise, she had no other specific risk factors (nonsmoker with no prior history of cardiorespiratory disease or diabetes mellitus and an admission BMI 21.9 kg/m^2^). Her husband had also suffered with similar symptoms for the preceding two weeks. On initial assessment in the emergency department, she was febrile at 38.5°C, tachycardic at 110 bpm with a stable blood pressure, and saturating 95% on room air. Her bloods were unremarkable, with only mild raised inflammatory markers, with no other abnormalities.

**Figure 1:**
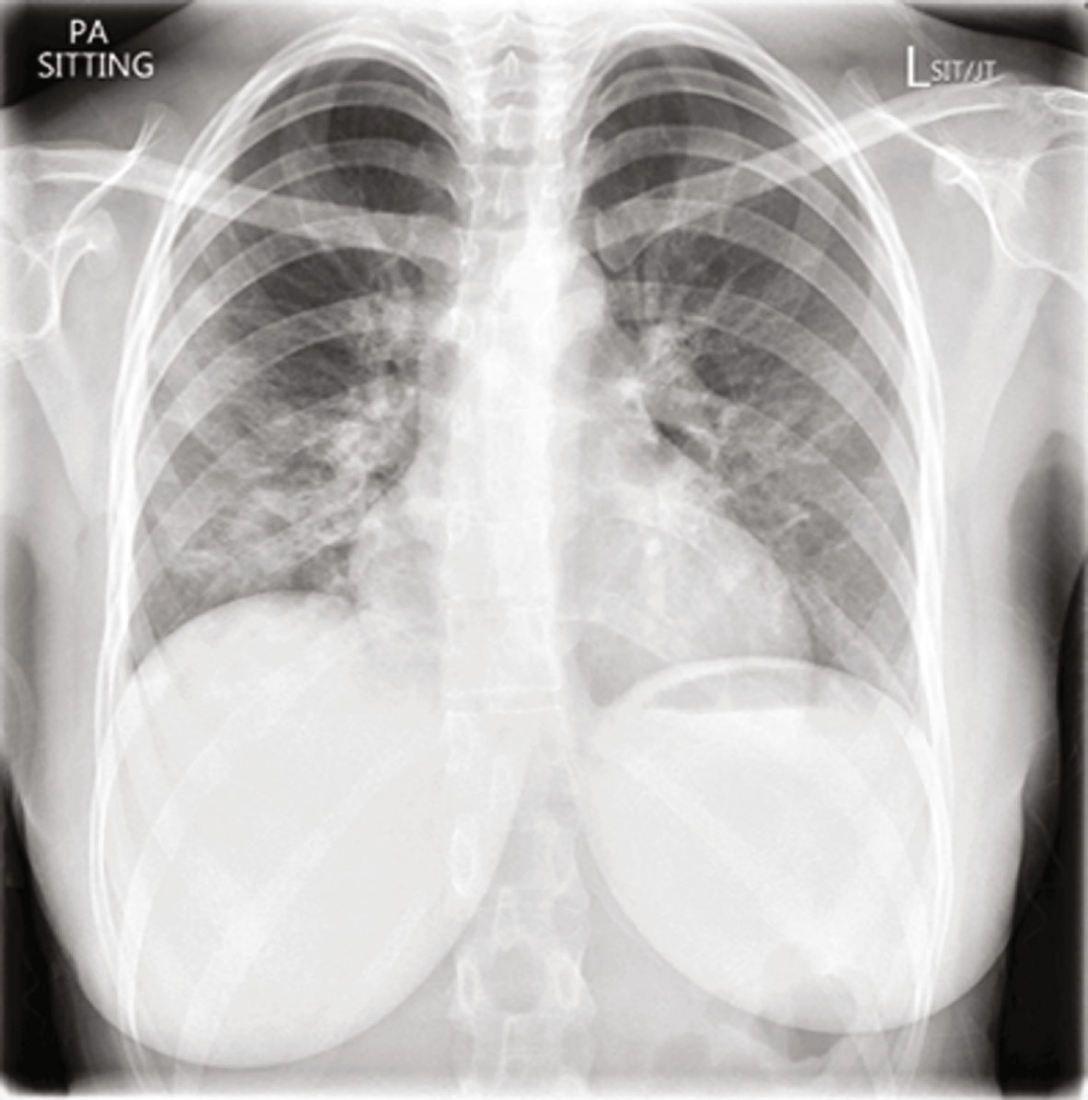
Admission chest radiograph in the emergency department.

An admission chest radiograph in the emergency department, at the University Hospital Southampton, demonstrated bilateral patchy areas of increased opacity and prominent lung markings (Figure 1), in keeping with the COVID-19 infection when taken in combination with the clinical picture.

**Figure 2:**
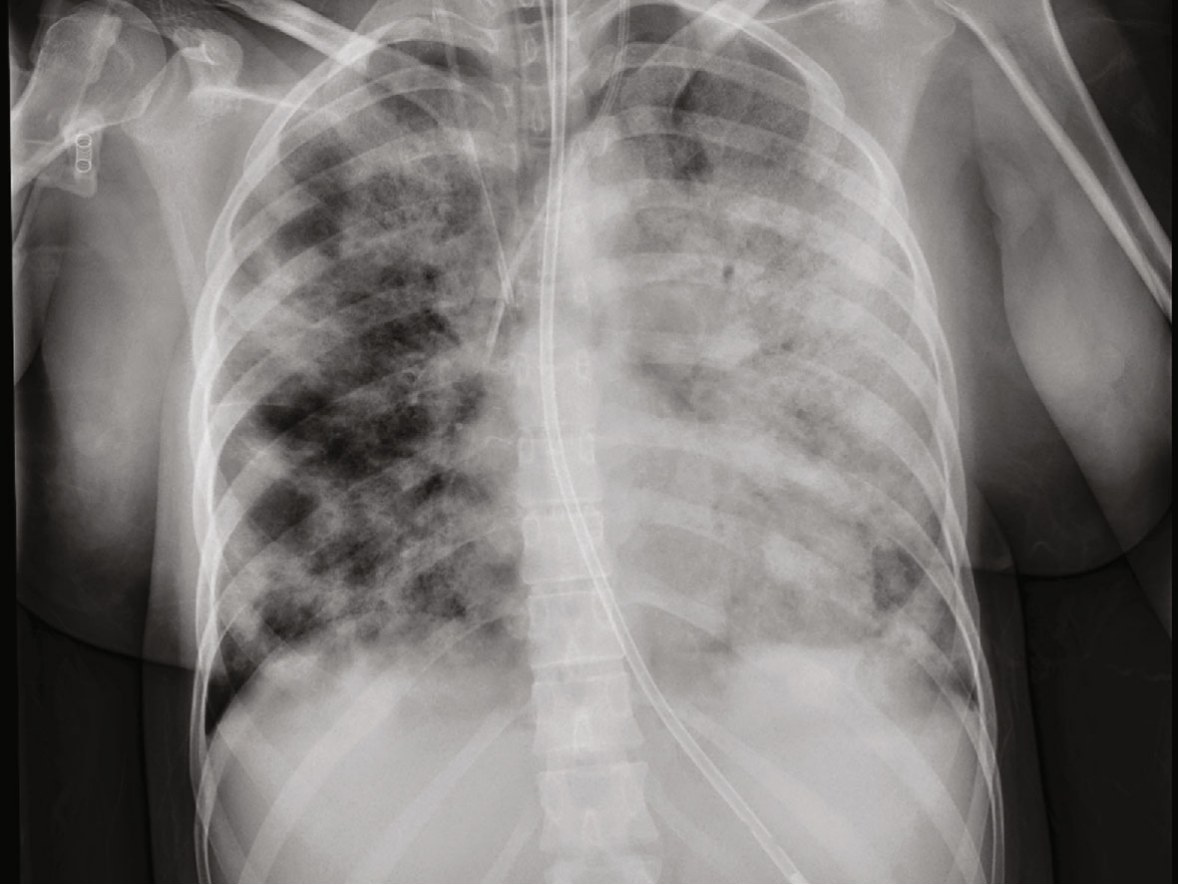
Chest radiograph on the general intensive care unit. Showing worsening of bilateral patchy changes which is worse on the left. Nasogastric tube, central line, and Vas Cath are noted.

She was admitted to the acute medical unit, but her respiratory function rapidly deteriorated, however, necessitating a trial of NIV. She quickly failed this trial and admitted to the intensive care unit (ICU), needing intubation and mechanical ventilation. She was confirmed positive on SARS-CoV-2 real-time reverse transcriptase polymerase chain reaction (RT-PCR) from a combined nose and throat swab. For this, the combined nose and throat swabs were transported in the VIROCULT virus transport medium. Samples were extracted and purified using the magnetic particle extraction on the Thermo Scientific KingFisher Flex. PCR amplification was performed on the Applied Biosystems (ABI) 7500 by using the Viasure SAR-CoV-2 RT-PCR kit, targeting the ORF1ab and N gene. Additionally, primers and probes for the World Health Organisation E gene assay (including an internal positive amplification control from extraction) was also used to enhance the sensitivity. A repeat chest radiograph showed considerable interval worsening of parenchymal opacification within both lungs (Figure 2).

She was mechanically ventilated and required different ventilation strategies, including multiple proning positions. She was referred for extracorporal membrane oxygenation (ECMO) support at a regional ECMO center. As she had been ventilated for more than ten days, it was felt that the patient would not benefit from ECMO at this later time point. Following some helpful advice from the ECMO center, she was started on a course of methylprednisolone (1 mg/kg, twice a day) for a week, and her ventilation mode was modified to airway pressure release ventilation (APRV).

This is an open lung ventilation mode with longer inspiratory times and can result in an increase in mean airway pressures to improve oxygenation. There was an initial improvement in her oxygenation, without any significant deterioration in arterial carbon dioxide concentration (PaCO_2_) or pH, and plateau pressures maintained around 30 cm of H_2_O. However, at day 14, the beneficial effect of APRV was not sustained, and she was switched back to a mandatory ventilation mode with adaptive pressure controlled tidal volume-targeted ventilation strategy and required continued paralysis and further prone positioning cycles.

**Figure 3:**
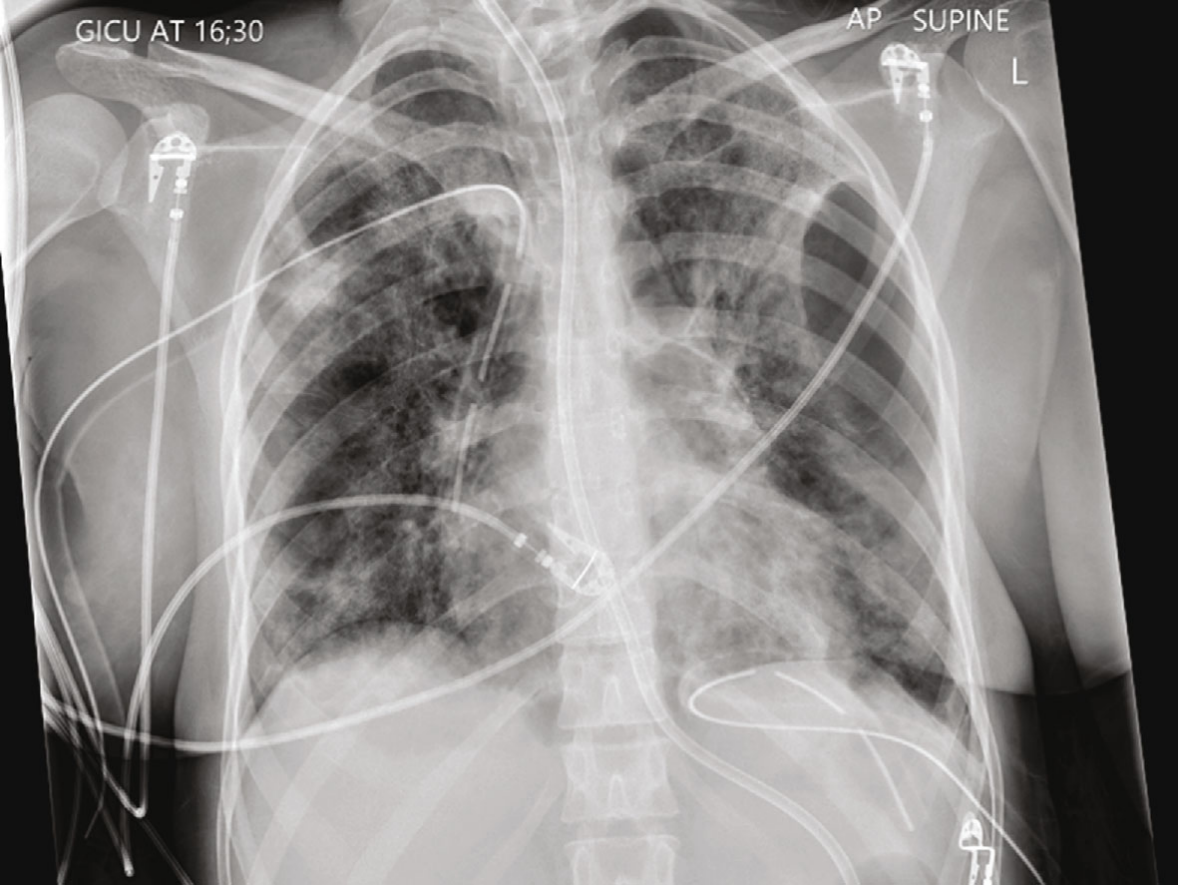
Chest radiograph and postinsertion of two intercostal chest drains with partial unresolved left-sided pneumothorax.

During one of her prone positioning episode on day 14 of her admission, her airway pressures increased dramatically with cardiovascular compromise. She was clinically diagnosed with tension pneumothorax. She was immediately placed supine and received a needle decompression, followed by insertion of bilateral intercostal chest drains (ICDs) (Figure 3), which improved her respiratory function but with some residual pneumothorax evident in the left hemithorax. The insertion of the ICDs was performed as an aseptic procedure with full personnel protective equipment (PPE), long sleeve gown, FFP3 respirator mask, and face and eye protection with full face visor and gloves. After this, she went on to have multiple ICDs, including an ICD under video-assisted guidance to place due to improve her complex hydropneumothorax. These ICDs ranged from 24-26 French and were placed on suction to aid resolution and drainage of the pneumothorax.

**Figure 4:**
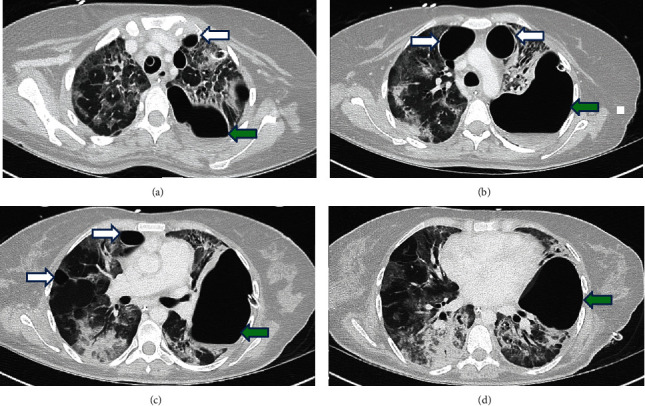
(a–d) Serial axial sections of thorax computed tomography (CT), showing bilateral ground glass opacification and pneumatoceles (white arrows) with large complex left hydropneumothorax (green arrows).

After a period of recovery and stability, no longer requiring any proning, she had a size 7.5 cuffed percutaneous tracheostomy placed at day 24. However, she was slow to progress in her respiratory wean. She did develop a swinging pyrexia with raised inflammatory markers, including a procalcitonin (PCT) of 6.4 *μ*g/L. A thorax CT scan demonstrated a large loculated left hydropneumothorax, bilateral anterior pneumatoceles, widespread bilateral ground glass, and crazy paving appearances with the radiological appearance of classic COVID-19 pneumonia (Figure 4). The pyrexia was likely related to the infected hydropneumothorax. There was no evidence of pulmonary embolism (PE) on her thorax CT scan. Development of PE formed a part of the initial differential diagnosis, as evidence is emerging of COVID-19 being a hypercoagulable state, with increased risk of venous thromboembolism (VTE). Her D-dimer was >5000 mmol/l persistently throughout the initial part of her admission, but this was likely related to the COVID-19 infection rather than the new VTE/PE. She also had a formal transthoracic echocardiogram, which demonstrated normal right and left ventricular function and preserved cardiac valves without any echocardiographic evidence of endocarditis.

Initially, she was considered too ill to undergo any surgical procedures, as she would have needed a thoracotomy with the possibility of a lobectomy or even a pneumonectomy with adhesiolysis to treat her complex hydropneumothorax. A multidisciplinary team (MDT) with thoracic anesthetists and thoracic surgeons opted for conservative management with ICDs to allow a period of time for rehabilitation. She has developed recurrent pneumothorax following the removal of her drains and required multiple ICDs throughout her admission. Pleurodeses were not contemplated at this stage as it would have hindered future definitive surgical interventions. However, despite the placement of multiple ICDs, serial thoracic CT scans showed persistent changes with an increase in the size of the left-sided hydropneumothorax. Following a prolonged course of antibiotics and continued intercostal drainage of her hydropneumothorax, she made a steady recovery in her physical status and showed improvement in her ventilator dependency. She underwent a video-assisted thoracoscopic surgery (VATS) with the washout of the hydropneumothorax and a further ICD placement on day 60.

Although she was treated with multiple courses of antibiotics for presumed infections including a catheter-related infection and ventilator-associated pneumonia, the primary infective focus was her infected hydropneumothorax, which has invariably hindered her weaning process. However, she was successfully treated with antimicrobials and steadily progressed with her respiratory wean. She also received daily routine physiotherapy sessions to improve her ICU-acquired weakness and facilitate a goal-directed respiratory wean to liberate her from the ventilator.

**Figure 5:**
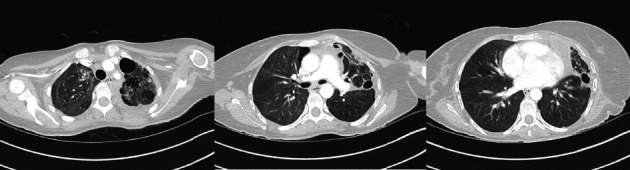
Serial axial sections of thorax computed tomography (CT) after tracheostomy decannulation showing bilateral residual scarring from the COVID-19 infection with significant improvement in the left hydropnemothorax and pneumatoceles.

After 109 days on ICU, she was successfully decannulated and stepped down to a respiratory ward, where she continued to receive the appropriate physiotherapy. She was discharged to a rehabilitation facility after a total of 116 days inpatient stay in the acute hospital and has been subsequently discharged home following an additional period of rehabilitation. Her most recent thorax CT showed significant improvement, with a large reduction in the size of her left hydropneumothorax (Figure 5). She is awaiting nonurgent outpatient follow-up with the COVID-19 clinic and thoracic surgeons.

## 3. Discussion

There have been several case reports to date of both pneumothorax and pneumomediastinum in COVID-19 patients that have either resolved with conservative management [4–7], chest drain insertion [8] or with bleb resection [3]. The underlying mechanism is unclear. Whether it is a combination of inflammatory injury from COVID-19 pneumonia and barotrauma has not been determined, as some patients received supplementary oxygen via facemask, NIV or high flow nasal oxygen (HFNO), and still develop a spontaneous pneumothorax. Viral pulmonary infections, such as H1N1 influenza, are rarely associated with spontaneous pneumomediastinum and pneumothorax. Suggested pathophysiology in these cases has been increased alveolar pressure through coughing or positive pressure ventilation and eventual alveolar damage [10].

In our patient, the cause for her recurrent pneumothoracies, hydropneumothorax and development of pneumatocoeles are unclear. Pneumothorax, pneumomediastinum, pleural and pericardial effusions, lymphadenopathy and cavitations are uncommon, but possible findings with COVID-19 disease progression [1, 3, 5–7], but again, pathophysiology remains unclear. The possible explanations include that the severe COVID-19 lung infection itself may lead to a chronic cystic lung disease state, or it may possibly be related to barotrauma and volutrauma as a result of the difficult mechanical ventilation pathway she endured. There were occasions where she had very high mean airway pressures and the use of APRV, which is a high-pressure ventilation strategy, may have caused barotrauma resulting in this atypical finding. However, it is surprising in a young patient with no significant past medical history or underlying lung pathology to develop such extensive, persistent, pulmonary changes. Thin-walled cystic changes were noted in young patients with COVID-19 lung infection following NIV and even without institution of mechanical ventilation, suggesting it may be a spectrum of the natural history of severe COVID-19 lung disease [11].

To the best of our knowledge, there are no other reported cases of recurrent pneumothoracies with large hydro-pneumothorax and pneumatoceles, in conjunction with the classical COVID-19 widespread bilateral GGO. There has been a single report of two patients that received non-invasive ventilation, developing a small pneumothorax and pulmonary cysts, <5 cm in size. These all reduced in size with CT follow up and needed no intervention or treatment [11]. Also, a report of a tension pneumothorax in a patient clinically suspected to have COVID-19 that resolved with insertion of a single ICD and no further complications [8].

The case reports for the two patients that needed bleb resection for their pneumothorax suggest procedure timing for thoracotomy is unclear and needs an MDT decision [3]. In their patients, a more aggressive and earlier operative attempt was planned and was successful. However, with our patient, due to the ongoing respiratory support and intermittent ongoing air leak, an MDT with thoracic surgeons and thoracic anesthetists was done, and the consensus decision was made that an early thoracotomy will be a high risk procedure and should be considered when she is recovered from her acute illness following a period of rehabilitation.

It is difficult to say if earlier surgical intervention could have prevented the multiple sequalae she suffered because of the hydropneumothorax, but ultimately, she may have not survived the surgery and she eventually responded well to conservative management. She remains our longest ventilated COVID-19 patient so far and was discharged home after a short duration of rehabilitation in a rehabilitation hospital.

We present a rare case and CT findings of a young woman, with no significant medical history or underlying lung pathology, who developed extensive bilateral pulmonary changes including a large, persistent hydropneumothorax and pneumatoceles following the COVID-19 infection. This is a rare complication of COVID-19 not previous reported in the literature, and it has had a significant impact on this patient's recovery, resulting in the delayed progress of her weaning from mechanical ventilatory support and a prolonged stay in the intensive care unit.

## Data Availability

No data sets were used for this manuscript.
